# Supplementary material for: Completely aqueous processable stimulus responsive organic room temperature phosphorescence materials with tunable afterglow color
Source: Nat Commun. 2022 Jan 17;13:347. doi: 10.1038/s41467-022-28011-6 (PMC8764117; doi:10.1038/s41467-022-28011-6)
Supplement: Supplementary file 1 — Supplementary Information [file 41467_2022_28011_MOESM1_ESM.pdf]

Supplementary information

Completely Aqueous Processable Stimulus Responsive Organic Room Temperature Phosphorescence Materials with Tunable Afterglow Color

Dan Li[1], Yujie Yang[1], Jie Yang[1]*, Manman Fang[1], Ben Zhong Tang[1,2]* and Zhen Li[1,3,4,5]*

[1]Institute of Molecular Aggregation Science, Tianjin University, Tianjin 300072, China. E-mail: jieyang2018@tju.edu.cn, lizhen@whu.edu.cn.

[2]Shenzhen Institute of Molecular Aggregate Science and Engineering, School of Science and Engineering, The Chinese University of Hong Kong, Shenzhen, 2001 Longxiang Boulevard, Longgang District, Shenzhen City, Guangdong 518172, China. E-mail: tangbenz@cuhk.edu.cn.

[3]Tianjin Key Laboratory of Molecular Optoelectronic Sciences, Department of Chemistry, Tianjin University, Tianjin 300072, China.

[4]Department of Chemistry, Wuhan University, Wuhan 430072, China.

[5]Joint School of National University of Singapore, Tianjin University, International Campus of Tianjin University, Binhai New City, Fuzhou 350207, China.

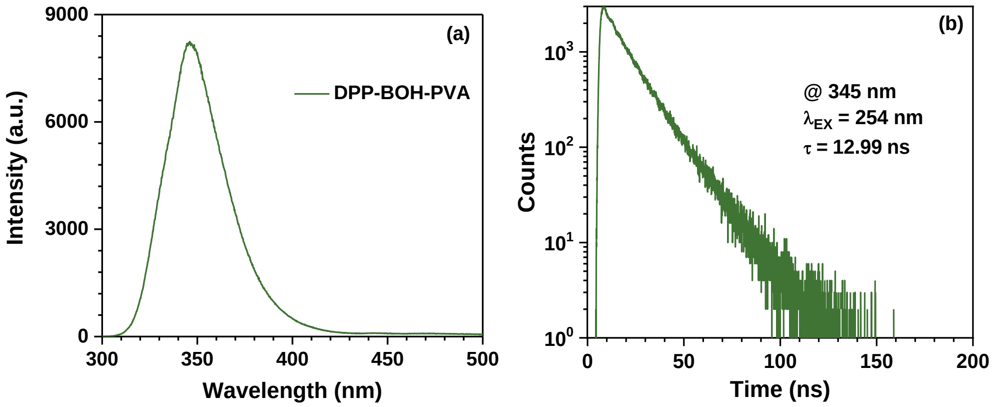

Supplementary Figure 1. a) The steady state spectrum and b) the time-resolved emission-decay profile of **DPP-BOH-PVA** film.

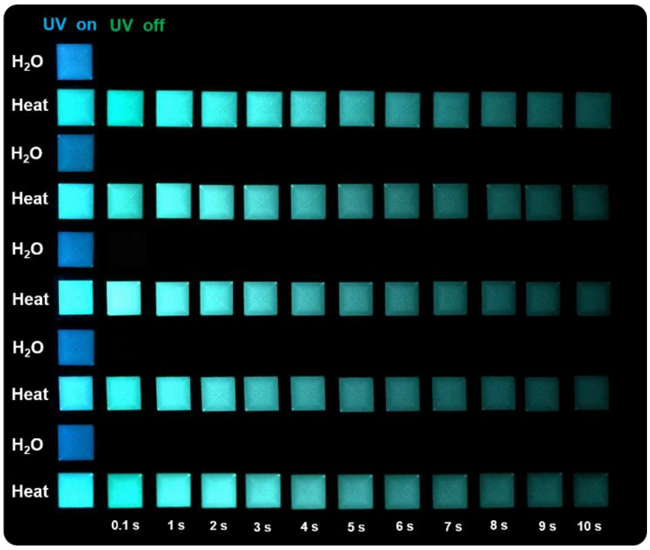

Supplementary Figure 2. Photographs of repeated cycles of the heating/water fuming processes for **DPP-BOH-PVA** film.

Supplementary Table 1. Phosphorescence lifetime of water-fumed **DPP-BOH-PVA**, **DPP-BOH-PVA-F** and **DPP-BOH-PVA-R** film after heating at different temperatures for 15 min.

	**DPP-BOH-PVA**	**DPP-BOH-PVA-F**	**DPP-BOH-PVA-R**
	τ_P (s) (475 nm)	τ_P (s) (475 nm, 533 nm)	τ_P (s) (475 nm, 581 nm)
H_2O	0.01	0.01, 0.00	0.01, 0.01
30 °C	0.81	1.40, 0.52	0.82, 0.55
40 °C	1.26	1.16, 0.65	1.11, 0.84
50 °C	1.54	1.43, 0.93	1.40, 1.11
60 °C	1.70	1.71, 0.99	1.70, 1.30
70 °C	2.22	2.30, 1.40	1.85, 1.45
80 °C	2.43	2.10, 1.60	2.20, 1.72

Supplementary Table 2. Photoluminescence quantum yields of **DPP-BOH-PVA**, **DPP-BO-PVA**, **DPP-BOH-PVA-C, DPP-BOH-PVA-F** and **DPP-BOH-PVA-R** films.

**DPP-BOH-PVA**		**DPP-BOH-PVA-F**		**DPP-BOH-PVA-R**	
Φ_F (%)	Φ_P (%)	$\Phi^{a)}$ (%)	$\Phi^{b)}$ (%)	$\Phi^{c)}$ (%)	$\Phi^{d)}$ (%)
17.60	7.51	13.28	51.68	15.89	60.88

$_F$ emission range for calculating quantum yield is from 325 to 430 nm.
$_P$ emission range for calculating quantum yield is from 430 to 650 nm.
[a)] emission range for calculating quantum yield is from 324 to 450 nm.
[b)] emission range for calculating quantum yield is from 450 to 700 nm.
[c)] emission range for calculating quantum yield is from 324 to 430 nm.
[d)] emission range for calculating quantum yield is from 430 to 700 nm.

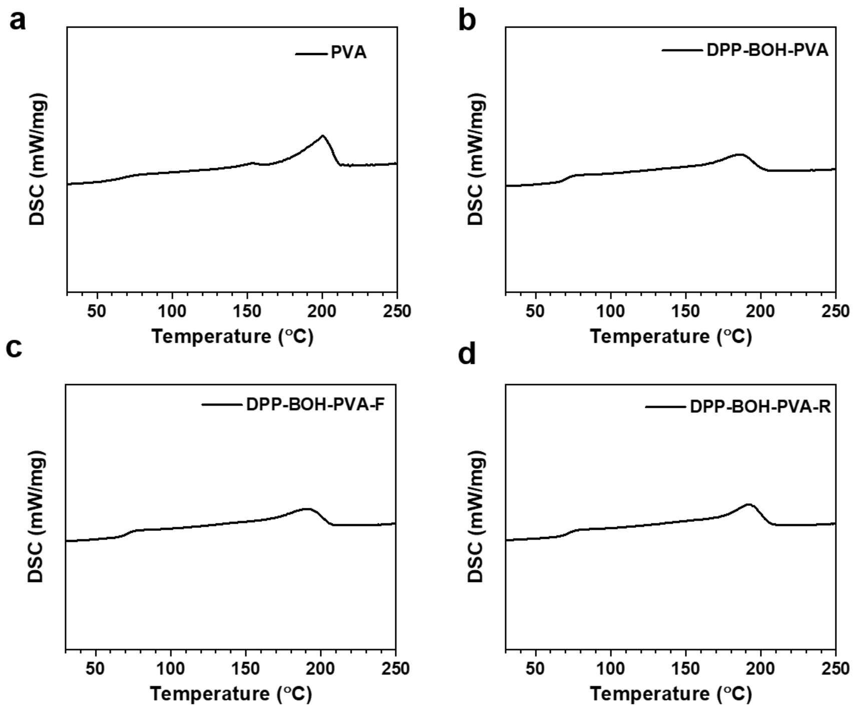

Supplementary Figure 3. The differential scanning calorimetry (DSC) curves of **PVA**, **DPP-BOH-PVA**, **DPP-BOH-PVA-F** and **DPP-BOH-PVA-R** films.

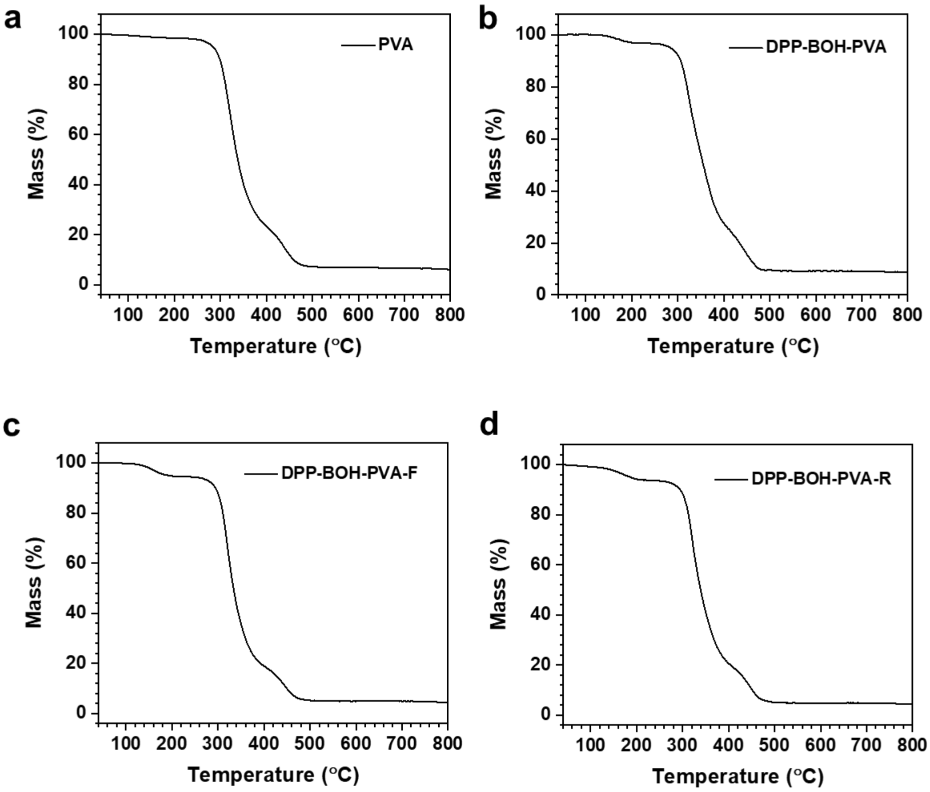

Supplementary Figure 4. The thermo-gravimetric analysis (TGA) curves of **PVA**, **DPP-BOH-PVA**, **DPP-BOH-PVA-F** and **DPP-BOH-PVA-R** films.

Supplementary Table 3. Glass transition temperatures and decomposition temperatures of **PVA**, **DPP-BOH-PVA**, **DPP-BOH-PVA-F** and **DPP-BOH-PVA-R** films.

	**PVA**	**DPP-BOH-PVA**	**DPP-BOH-PVA-F**	**DPP-BOH-PVA-R**
Glass transition temperature/°C	59.3	66.5	66.7	66.8
Decomposition temperature/°C	308.5	308.5	324.6	323.7

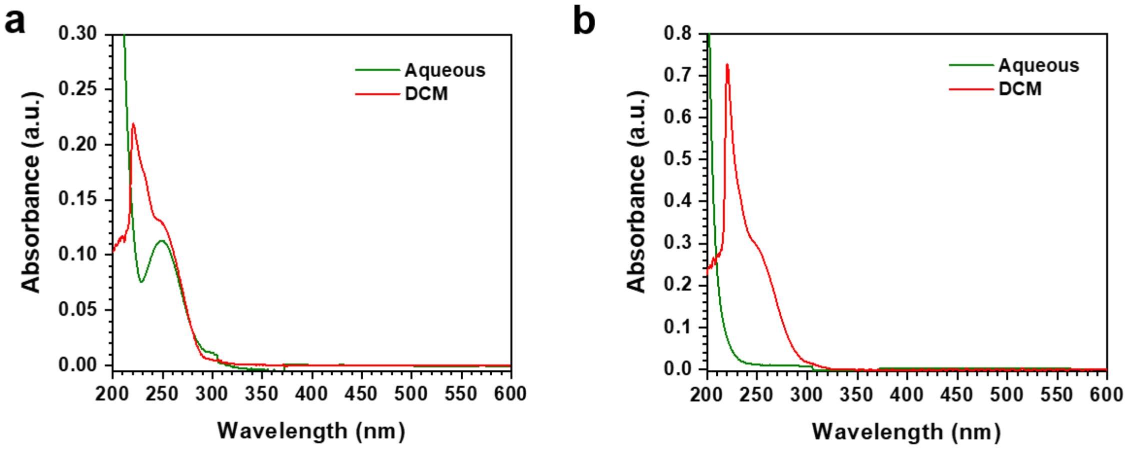

Supplementary Figure 5. The UV-vis absorption spectra of queous phase (green line) and dichloromethane (DCM) solution (red line) for a) **DPP-BOH-PVA** and b) **DPP-BO-PVA** after extracting their prepared aqueous solutions by dichloromethane (DCM) with the volume ratio of 1:1.

The reaction yield of **DPP-BOH-PVA** was calculated based on following equation:

$$A=\lg(1/T)=Kbc \qquad \text{(a)}$$

Where A, T, K, b and c are absorbance, transmittance, molar absorption coefficient, the distance the light travels in the sample and the concentration of the solution. The absorbance of aqueous phase and DCM solution after extraction of **DPP-BOH-PVA** is 0.113 and 0.130, respectively.

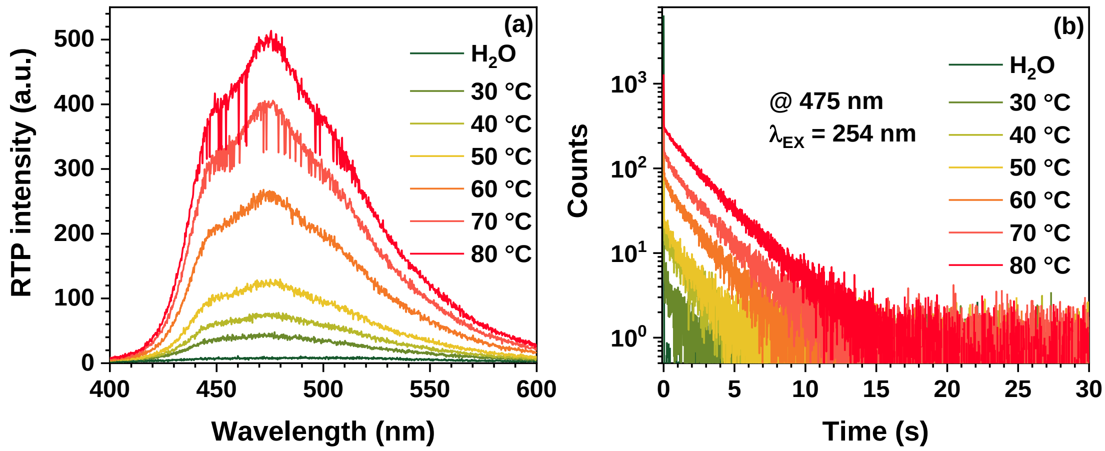

Supplementary Figure 6. a) Phosphorescence spectra and b) corresponding decay curves of water-fumed **DPP-BOH-PVA-C** film after heating at different temperatures for 15 min.

Supplementary Table 4. Phosphorescence lifetime of water-fumed **DPP-BOH-PVA-C** film after heating at different temperatures for 15 min.

H_2O	30 °C	40 °C	50 °C	60 °C	70 °C	80 °C
0.01 s	0.01 s	0.01 s	0.04 s	0.13 s	0.24 s	0.48 s

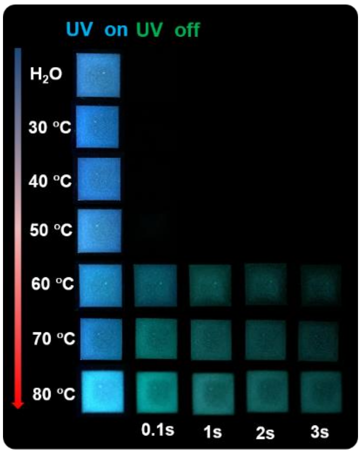

Supplementary Figure 7. Photographs of water-fumed **DPP-BOH-PVA-C** films after heating at different temperatures (30 °C-80 °C). The temperature gradient was 10 °C and the corresponding heating time was 15 min, after which the RTP behaviors were studied when the samples were cooled to room temperature.

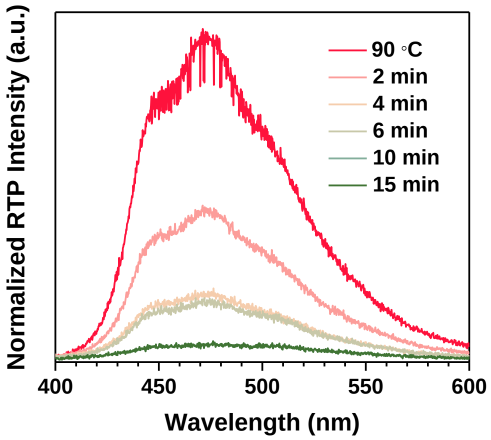

Supplementary Figure 8. Phosphorescence spectra of desiccative **DPP-BOH-PVA-C** film under water fuming for different times.

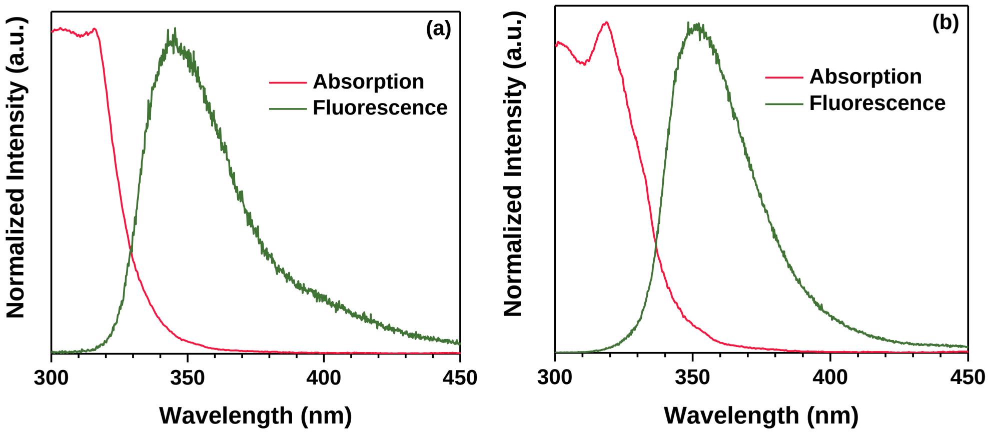

Supplementary Figure 9. The steady-state PL spectra and the UV-vis absorption spectra of a) **DPP-BO-PVA** and b) **DPP-BOH-PVA-C**.

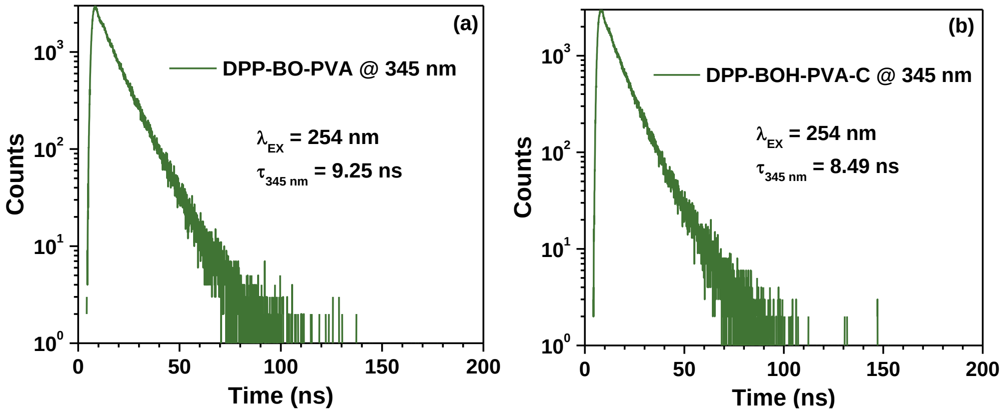

Supplementary Figure 10. Time resolved emission-decay profiles of a) **DPP-BO-PVA** (@ 345nm) and b) **DPP-BOH-PVA** (@345 nm).

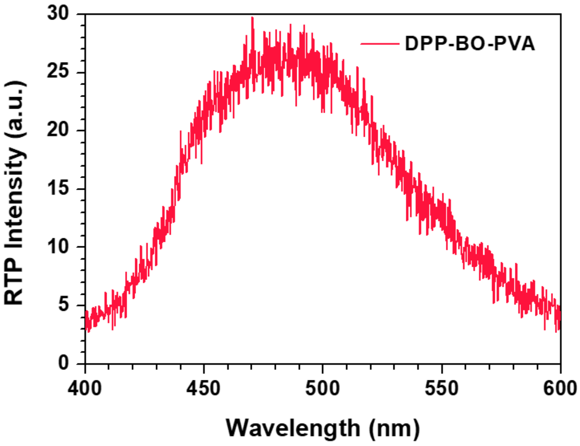

Supplementary Figure 11. Phosphorescence spectrum of **DPP-BO-PVA** film after heating.

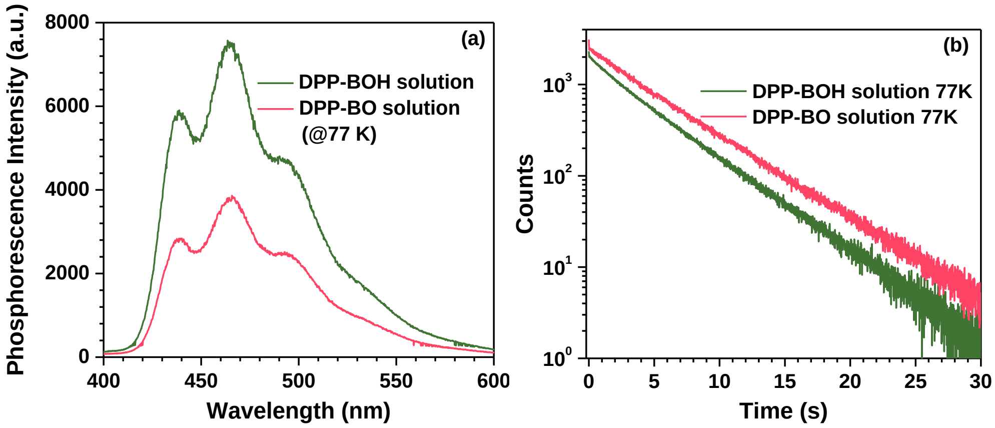

Supplementary Figure 12. a) Phosphorescence spectra and b) time resolved emission-decay profiles of **DPP-BOH** and **DPP-BO** in tetrahydrofuran (THF) solution at 77 K.

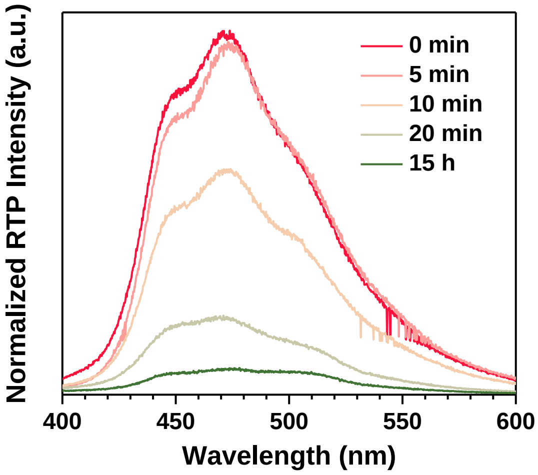

Supplementary Figure 13. RTP spectra of **DPP-BOH-PVA** film after putting in oxygen for different times.

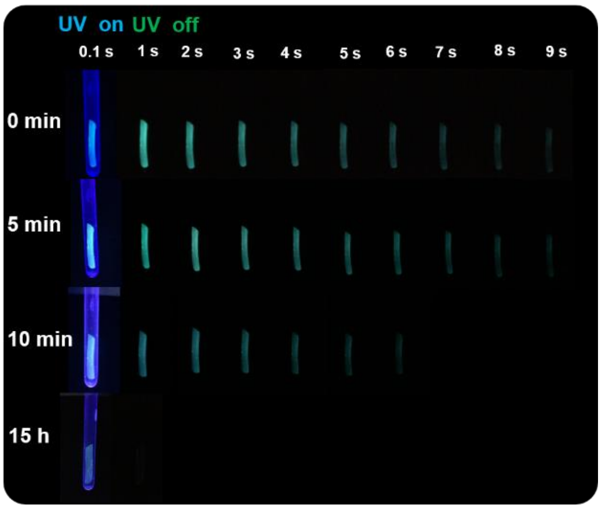

Supplementary Figure 14. Photographs of **DPP-BOH-PVA** films after filling with oxygen for different times.

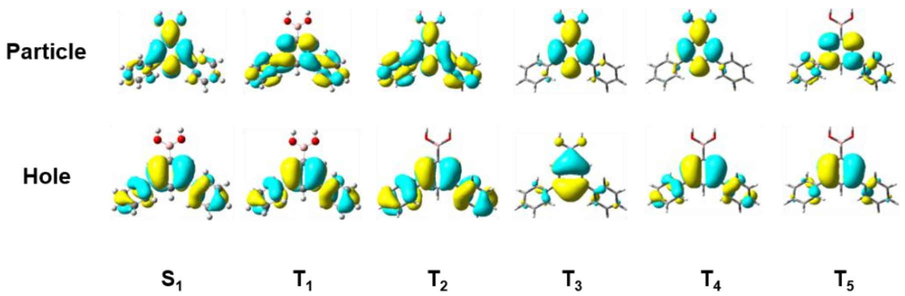

Supplementary Figure 15. The theoretical calculations about natural transition orbitals (NTOs) for **DPP-BOH**.

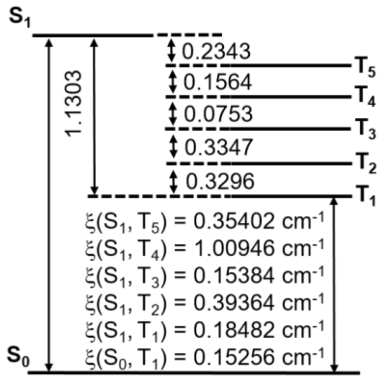

Supplementary Figure 16. Calculated energy diagram and spin-orbit coupling (SOC) value (ξ) of **DPP-BOH**.

Supplementary Table 5. Dynamic photophysical parameters of **DPP-BOH-PVA**.

τ_f/ns	τ_p/s	Φ_f/%	Φ_p/%	$k_{f,r}$/s^{-1}	Φ_{isc}/%	k_{isc}/s^{-1}	$k_{p,r}$/s^{-1}	$k_{p,nr}$/s^{-1}	f
12.99	2.43	17.60	7.51	1.35×10^7	82.40	6.34×10^7	0.04	0.37	0.0078

The dynamic photophysical parameters were calculated based on following equations:

$k_{f,r} = \Phi_f / \tau_f$ (b)

$\Phi_{isc} = 1\text{-}\Phi_f\text{-}\Phi_{ic} \approx 1\text{-}\Phi_f$ (c)

$\tau_f = 1/(k_{f,r}+ k_{f,nr}+k_{isc})$;

$\Phi_{isc} = k_{isc}/(k_{f,r}+ k_{f,nr}+k_{isc}) = k_{isc}\times \tau_f$;

$k_{isc} = \Phi_{isc}/ \tau_f$ (d)

$\tau_p = 1/(k_{p,r}+ k_{p,nr})$;

$\Phi_p = (\Phi_{isc}\times k_{p,r})/(k_{p,r}+k_{p,nr}) = \Phi_{isc}\times k_{p,r}\times \tau_p$;

$k_{p,r} = \Phi_p/(\Phi_{isc}\times \tau_p)$ (e)

$k_{p,nr} = 1/\tau_p\text{-}k_{p,r}$ (f)

$k_r = fE_{vert}^2/1.499$ (g)

$E_{vert} = 1240/\lambda_p$ (h)

Where, $k_{f,r}$, k_{isc} , $k_{p,r}$, $k_{p,nr}$, f, E_{vert}, k_r, λ_P are the radiative rate constant of prompt fluorescence, rate constant of intersystem crossing (ISC), radiative rate constant of phosphorescence, non-radiative rate constant of phosphorescence, the oscillator strength, vertical excitation energy, spontaneous radiation rate and first RTP emission peak.

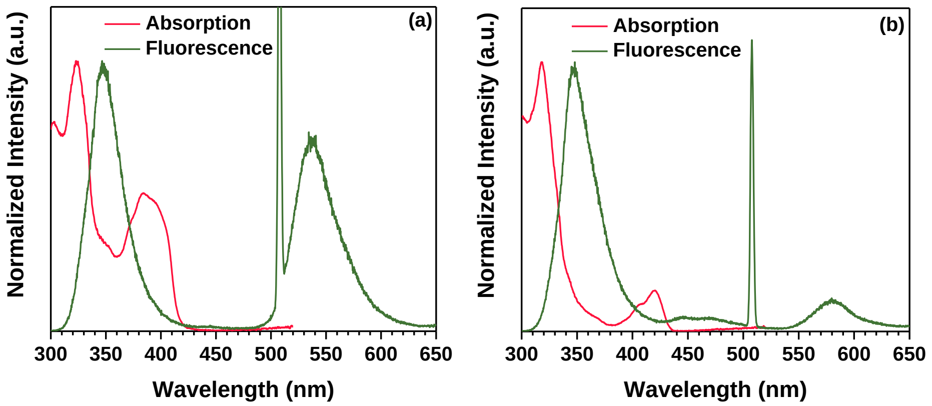

Supplementary Figure 17. The steady-state spectra and the UV-vis absorption spectra of a) **DPP-BOH-PVA-F** and b) **DPP-BOH-PVA-R**.

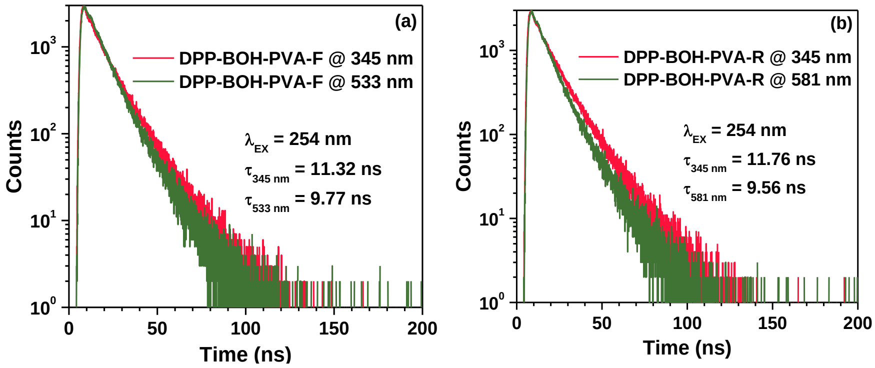

Supplementary Figure 18. Time resolved emission-decay profiles of a) **DPP-BOH-PVA-F** (@345nm and 533 nm) and b) **DPP-BOH-PVA-R** (@345nm and 581 nm).

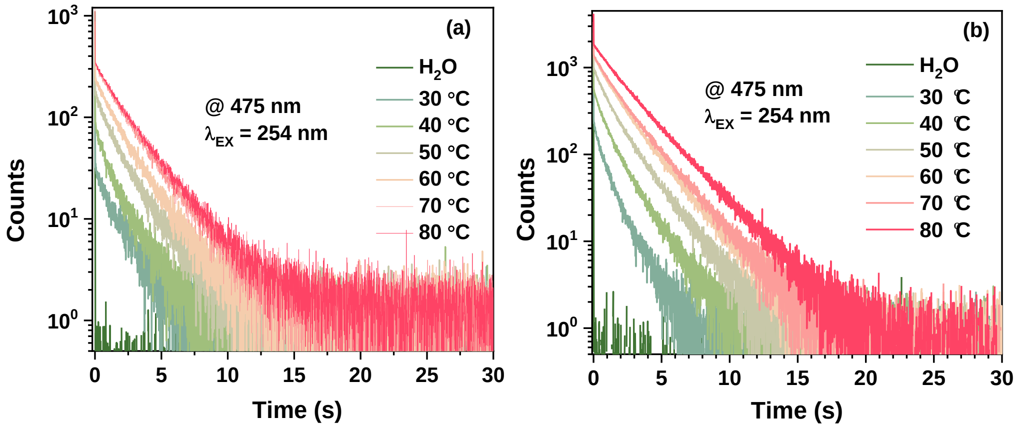

Supplementary Figure 19. Time resolved emission-decay profiles of a) **DPP-BOH-PVA-F** (@475 nm) and b) **DPP-BOH-PVA-R** (@475 nm).

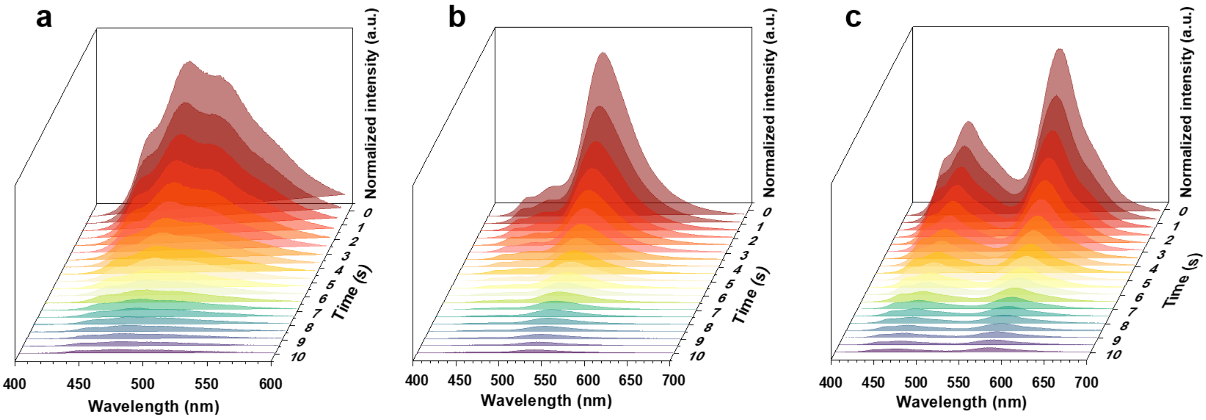

Supplementary Figure 20. Time-resolved phosphorescence spectra after turning off the 254 nm UV lamp of **a**) **DPP-BOH-PVA**, **b**) **DPP-BOH-PVA-F** and **c**) **DPP-BOH-PVA-R** film.

Supplementary Table 6. Calculated rate constant and efficiency of FRET from **DPP-BOH-PVA** to fluorescein and rhodamine B.

	$E_{S\to S}$/%	$E_{T\to S}$/%	$\tau_{0,D}$/s	k_{ET}/s^{-1}
**DPP-BOH-PVA-F**	12.86	13.58	2.43	0.06
**DPP-BOH-PVA-R**	13.32	9.47		0.04

The rate constant of FRET was calculated using the following equations:

$k_{ET}(R) = (R_0/R)^6/\tau_{0,D}$ (i)

$E = R_0^6/(R_0^6+R^6)$ (j)

Where k_{ET}, $\tau_{0,D}$, E, R and R_0 are the rate constant of FRET, the lifetime of donor without acceptor, the efficiency of FRET, the transfer radius and the critical transfer radius where FRET occurs effectively.

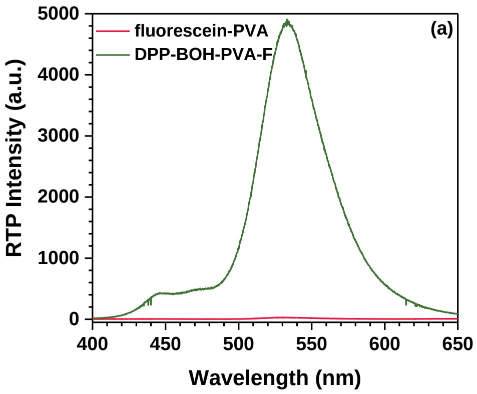

Supplementary Figure 21. a) Phosphorescence spectra of **PVA** doped fluorescein (**fluorescein-PVA**) and **DPP-BOH-PVA-F** films after heating. **b)** Phosphorescence spectra of **PVA** doped rhodamine (**rhodamine-PVA**) and **DPP-BOH-PVA-R** films after heating.

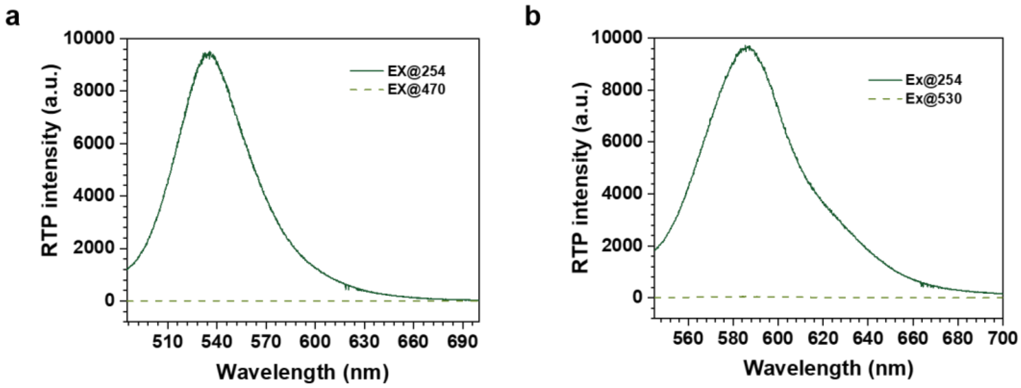

Supplementary Figure 22. Phosphorescence spectra of **a**) **DPP-BOH-PVA-F** film and **b**) **DPP-BOH-PVA-R** film at different excitation wavelengths.

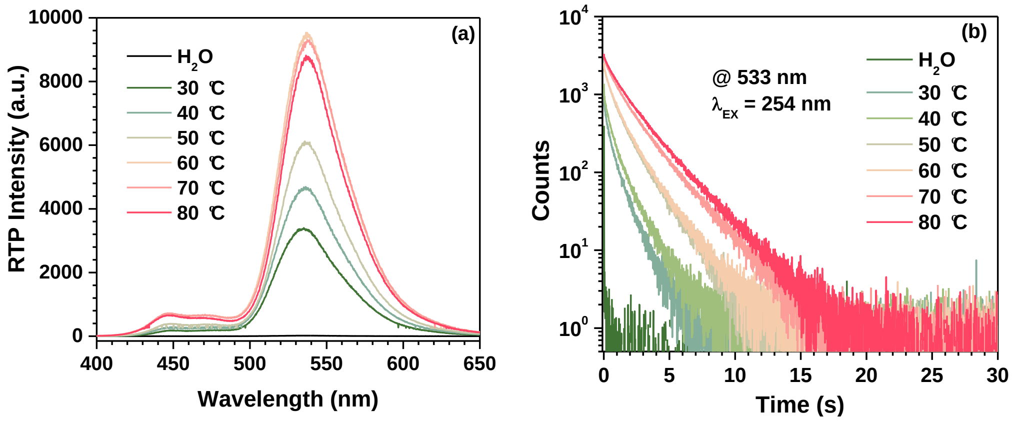

Supplementary Figure 23. a) RTP spectra of water-fumed **DPP-BOH-PVA-F** film after heating at different temperatures for 15 min. b) Time-resolved emission-decay profiles of water-fumed **DPP-BOH-PVA-F** film after heating for at different temperatures for 15 min.

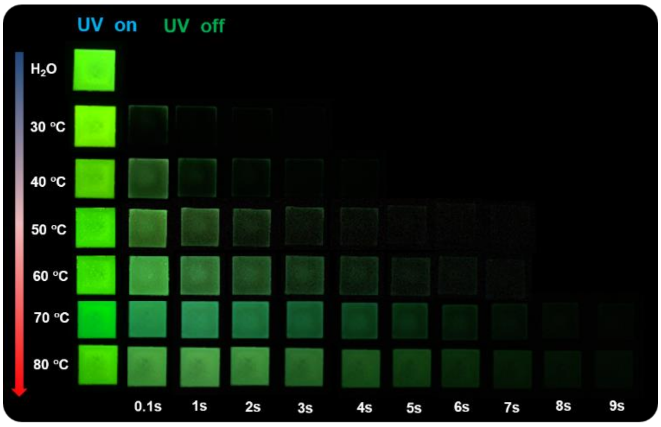

Supplementary Figure 24. Photographs of water-fumed **DPP-BOH-PVA-F** films after heating at different temperatures (30 °C-80 °C). The temperature gradient was 10 °C and the corresponding heating time was 15 min, after which the RTP behaviors were studied when the samples were cooled to room temperature.

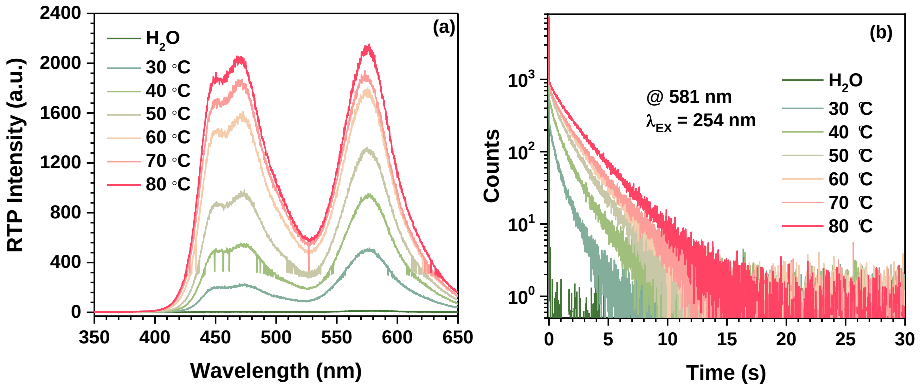

Supplementary Figure 25. a) RTP spectra of water-fumed **DPP-BOH-PVA-R** film after heating at different temperatures for 15 min. b) Time-resolved emission-decay profiles of water-fumed **DPP-BOH-PVA-R** film after heating for at different temperatures for 15 min.

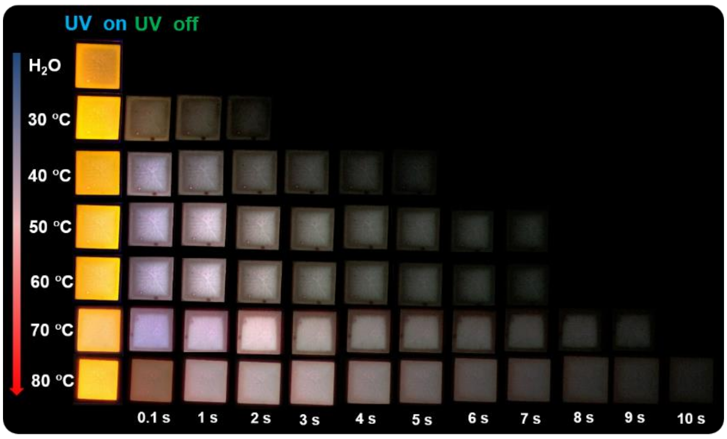

Supplementary Figure 26. Photographs of water-fumed **DPP-BOH-PVA-R** films after heating at different temperatures (30 °C-80 °C). The temperature gradient was 10 °C and the corresponding heating time was 15 min, after which the RTP behaviors were studied when the samples were cooled to room temperature.

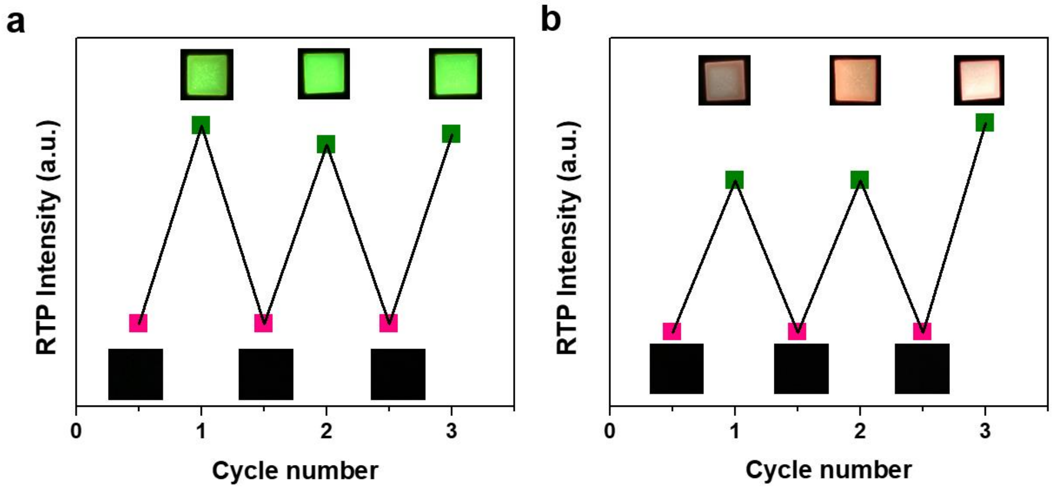

Supplementary Figure 27. Repeated cycles of the heating/water fuming processes and the corresponding photographs of a) **DPP-BOH-PVA-F** film and b) **DPP-BOH-PVA-R** film after turning off the UV lamp.

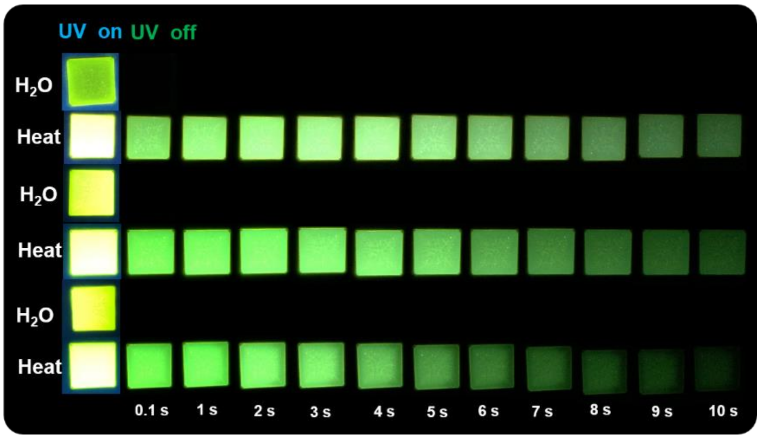

Supplementary Figure 28. Photographs of repeated cycles of the heating/water fuming processes of **DPP-BOH-PVA-F** film.

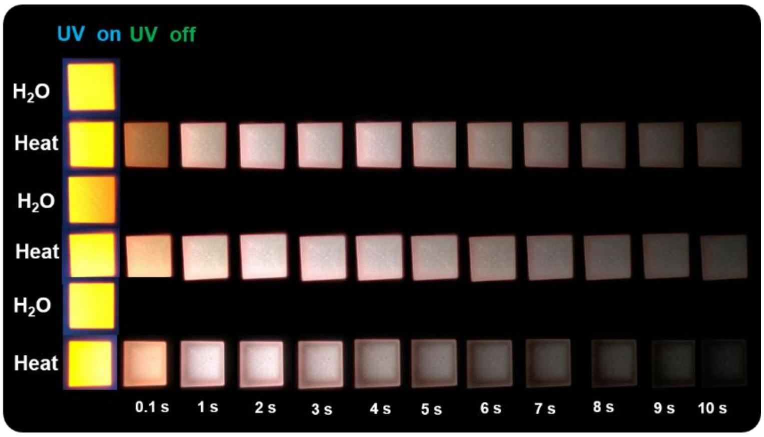

Supplementary Figure 29. Photographs of repeated cycles of the heating/water fuming processes of **DPP-BOH-PVA-R** film.

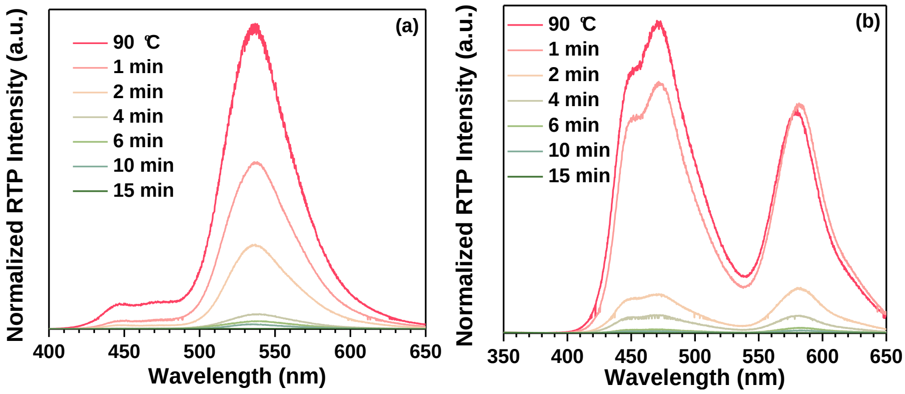

Supplementary Figure 30. RTP spectra of desiccative a) **DPP-BOH-PVA-F** film and b) **DPP-BOH-PVA-R** film under water fuming for different times.

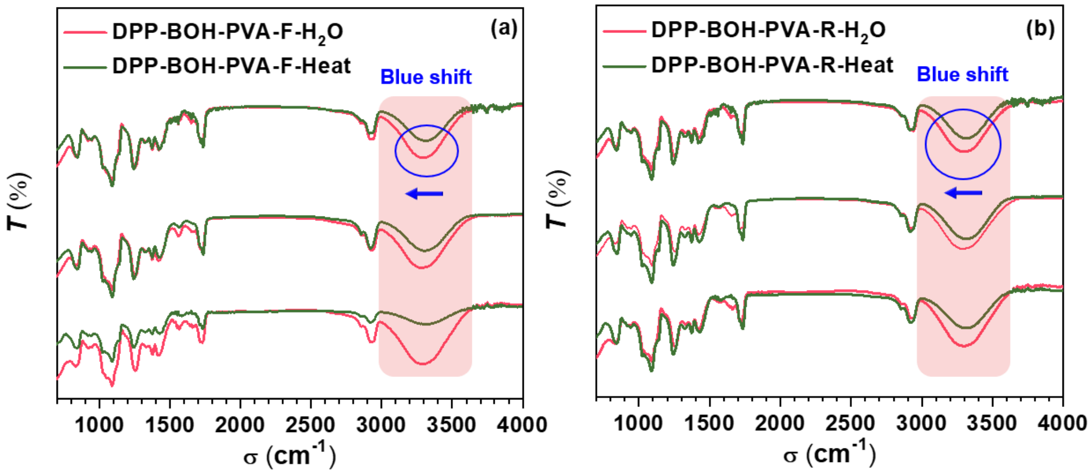

Supplementary Figure 31. Three repeated cycles for the Fourier transform infrared (FTIR) spectra of a) **DPP-BOH-PVA-F** film and b) **DPP-BOH-PVA-R** film under of the heating/water fuming stimuli.

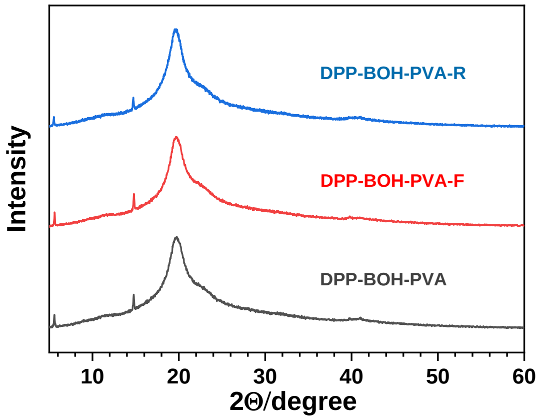

Supplementary Figure 32. Powder X-ray diffraction (XRD) patterns of **DPP-BOH-PVA**, **DPP-BOH-PVA-F** and **DPP-BOH-PVA-R.**
