# Supplementary material for: Completely aqueous processable stimulus responsive organic room temperature phosphorescence materials with tunable afterglow color
Source: Nat Commun. 2022 Jan 17;13:347. doi: 10.1038/s41467-022-28011-6 (PMC8764117; doi:10.1038/s41467-022-28011-6)
Supplement: Supplementary file 3 — Description of Additional Supplementary Files [file 41467_2022_28011_MOESM3_ESM.pdf]

Description of additional supplementary data files

Supplementary Movie S1. The video of heated DPP-BOH-PVA film before and after turning off the UV lamp with the 254 nm excitation.

Supplementary Movie S2. The video of heated DPP-BOH-PVA-F film before and after turning off the UV lamp with the 254 nm excitation.

Supplementary Movie S3. The video of heated DPP-BOH-PVA-R film before and after turning off the UV lamp with the 254 nm excitation.
